# Supplementary material for: Improvement of nuclear facilities DNP3 protocol data transmission security using super encryption BRC4 in SCADA systems
Source: PeerJ Comput Sci. 2021 Nov 4;7:e727. doi: 10.7717/peerj-cs.727 (PMC8594588; doi:10.7717/peerj-cs.727)
Supplement: Supplemental Information 4 [file peerj-cs-07-727-s004.pdf]

Appendix 1 The Time Complexity Analysis for Encryption Code

Code	Ci	∑Ci	Total
tic;	C1	1	C1
jum=length(data);	C3	1	C3
D=data(1);	C3	1	C3
for i=2:jum	C4	(n-1)	(n-1) C4
D=[D ';' data(i)];	C1	(n-1)	(n-1) C1
end;			
D=[D ';'];	C1	1	C1
H=[];	C1	1	C1
n=length(D);	C3	1	C3
for i=1:n	C4	n	nC4
H=[H char(D(i))];	C2	n	nC2
end;			
hasil=double(H);	C3	1	C3
kunci=randi(256,1,pk);	C3	3	3C3
n=length(hasil);	C3	1	C3
m=length(kunci);	C3	1	C3
if n>m	C5	1	C5
t=m+1;	C2	1	C2
KB=kunci;	C1	1	C1
while t<=n	C4	n	nC4
KB(t)=mod((KB(t-m)+KB(t-1)),256);	C2	6n	6nC2
t=t+1;	C2	1	C2
end;			
else			
KB=kunci(1:n);			
end;			
CB=[];	C1	1	C1
for j=1:n	C4	n	nC4
hasil2=hasil(j)-KB(j);	C2	n	nC2
CB=[CB mod(hasil2,256)];	C2	3	3C2
end;			
m=length(kunci);	C3	1	C3
if m<256	C5	1	C5
t=m+1;	C2	1	C2
KRC4=kunci;	C1	1	C1
while t<=256	C4	256	256C4
KRC4(t)=mod((KRC4(t-m)+KRC4(t-1)),256);	C2	6x256	1536C2
t=t+1;	C2	256	256C2
end;			
else			
KRC4=kunci(1:256);			

Code	Ci	∑Ci	Total
end;			
for i=1:256	C4	256	256C4
s(i)=i-1;	C2	256	256C2
end;			
j=0;	C1	1	C1
for i=1:256	C4	256	256C4
j=mod((j+s(i)+ KRC4(i)),256);	C2	5x256	1280C2
dummy=s(i);	C1	1	C2
s(i)=s(j+1);	C2	1	C2
s(j+1)=dummy;	C2	1	C2
end;			
i=0;	C1	1	C1
j=0;	C1	1	C1
for idx=1:256	C4	256	256C4
i =mod((i + 1),256) ;	C2	4x256	1024C2
j =mod((j + KRC4(i + 1)),256);	C2	5x256	1280C2
dummy=KRC4(i+1);	C2	1	C2
KRC4(i+1)=KRC4(j+1);	C2	2	2C2
KRC4(j+1)=dummy;	C2	1	C2
t=mod((KRC4(i + 1) + KRC4(j + 1)),256);	C2	6	6C2
PRC4(idx)=KRC4(t + 1) ;	C2	1	C2
end;			
if n<256	C5	1	C5
PRC4=PRC4(1:n);			
STL=mod((CB+PRC4),256);			
Else			
blok=ceil(n/256);	C3	1	C3
STL=[];	C1	1	C1
for i=1:blok	C4	n/256	nC4/256
if n>256	C5	n/256	nC5/256
dumi=CB(1:256);	C2	n/256	nC2/256
hasil3=mod((dumi+PRC4),256);	C2	4n/256	4nC2/256
KRC4=PRC4;	C1	n/256	nC1/256
for i=1:256	C4	n/256 x 256	nC4
s(i)=i-1;	C2	n	nC2
end;			
j=0;	C1	n/256	nC1/256
for i=1:256	C4	n/256 x 256	nC4
j=mod((j+s(i)+ KRC4(i)),256);	C2	5n	5nC2
dummy=s(i);	C1	n	nC1
s(i)=s(j+1);	C2	n	nC2
s(j+1)=dummy;	C2	n	nC2
end;			

Code	Ci	∑Ci	Total
i=0;	C1	n/256	nC1/256
j=0;	C1	n/256	nC1/256
for idx=1:256	C4	n/256 x 256	nC4
i =mod((i + 1),256) ;	C2	4n	4nC2
j =mod((j + KRC4(i + 1)),256);	C2	5n	5nC2
dummy=KRC4(i+1);	C2	n	nC2
KRC4(i+1)=KRC4(j+1);	C2	n	nC2
KRC4(j+1)=dummy;	C2	n	nC2
t=mod((KRC4(i + 1) + KRC4(j + 1)),256);	C2	6n	6nC2
PRC4(idx)=KRC4(t + 1) ;	C2	n	nC2
end;			
CB=CB(257:end);	C2	n/256	nC2/256
else			
PRC4=PRC4(1:n);			
hasil3=mod((CB+PRC4),256);			
end;			
STL=[STL hasil3];	C2	n/256	nC2/256
n=length(CB);	C3	n/256	nC3/256
end;			
end;			
cipher=[STL kunci pk];	C2	2	2C2
time=toc;	C1	1	C1
Te=time;	C1	1	C1
