# Supplementary material for: Improvement of nuclear facilities DNP3 protocol data transmission security using super encryption BRC4 in SCADA systems
Source: PeerJ Comput Sci. 2021 Nov 4;7:e727. doi: 10.7717/peerj-cs.727 (PMC8594588; doi:10.7717/peerj-cs.727)
Supplement: Supplemental Information 5 [file peerj-cs-07-727-s005.pdf]

Appendix 2 The Time Complexity Analysis for Decryption Code

**Code**	**Ci**	**∑Ci**	**Total**
tic;	C1	1	C1
pk=cipher(end);	C3	1	C3
n=length(cipher);	C3	1	C3
kunci=cipher(n-pk:end-1);	C2	2	2C2
m=n-pk-1;	C2	2	2C2
STL=cipher(1:m);	C2	1	C2
if pk<256	C5	1	C5
t=pk+1;	C2	1	C2
KRC4=kunci;	C1	1	C1
while t<=256	C4	256	256C4
KRC4(t)=mod((KRC4(t-pk)+KRC4(t-1)),256);	C2	6x256	1536C2
t=t+1;	C2	256	256C2
end;			
else			
KRC4=kunci(1:256);			
end;			
n=length(STL);	C3	1	C3
for i=1:256	C4	256	256C4
s(i)=i-1;	C2	256	256C2
end;			
j=0;	C1	1	C1
for i=1:256	C4	256	256C4
j=mod((j+s(i)+ KRC4(i)),256);	C2	5x256	1280C2
dummy=s(i);	C1	256	256C1
s(i)=s(j+1);	C2	256	256C2
s(j+1)=dummy;	C2	256	256C2
end;			
i=0;	C1	1	C1
j=0;	C1	1	C1
for idx=1:256	C4	256	256C4
i =mod((i + 1),256) ;	C2	4x256	1024C2
j =mod((j + KRC4(i + 1)),256);	C2	5x256	1280C2
dummy=KRC4(i+1);	C2	256	256C2
KRC4(i+1)=KRC4(j+1);	C2	2x256	1024C2
KRC4(j+1)=dummy;	C2	256	256C2
t=mod((KRC4(i + 1) + KRC4(j + 1)),256);	C2	6x256	1536C2
PRC4(idx)=KRC4(t + 1) ;	C2	256	256C2
end;			

**Code**	**Ci**	**∑Ci**	**Total**
if n<256	C5	1	C5
PRC4=PRC4(1:n);			
CB=mod((STL-PRC4),256);			
else			
blok=ceil(n/256);	C3	2	2C3
CB=[];	C1	1	C1
for i=1:blok	C4	n/256	n/256C4
if n>256	C5	n/256	n/256C5
dumi=STL(1:256);	C3	n/256	n/256C3
hasil=mod((dumi-PRC4),256);	C2	4n/256	4n/256C2
KRC4=PRC4;			
for I=1:256	C4	n	nC4
s(I)=I-1;	C2	n	C2
end;			
J=0;	C1	n/256	nC1/256
for I=1:256	C4	n	nC4
J=mod((J+s(I)+ KRC4(I)),256);	C2	5n	5nC2
dummy=s(I);	C1	n	C1
s(I)=s(J+1);	C2	n	nC2
s(J+1)=dummy;	C2	n	nC2
end;			
I=0;	C1	n/256	nC1/256
J=0;	C1	n/256	nC1/256
for idx=1:256	C4	n	nC4
I =mod((I + 1),256) ;	C2	4n	4nC2
J =mod((J + KRC4(I + 1)),256);	C2	5n	5nC2
dummy=KRC4(I+1);	C2	n	nC2
KRC4(I+1)=KRC4(J+1);	C2	2n	2nC2
KRC4(J+1)=dummy;	C2	n	nC2
t=mod((KRC4(I + 1) + KRC4(J + 1)),256);	C2	6n	6nC2
PRC4(idx)=KRC4(t + 1) ;	C2	n	nC2
end;			
STL=STL(257:end);	C3	n/256	nC3/256
else			
PRC4=PRC4(1:n);			
hasil=mod((STL-PRC4),256);			
end;			
CB=[CB hasil];	C2	1	C2
n=length(STL);	C3	1	C3

Code	Ci	∑Ci	Total
end;			
end;			
n=length(CB);	C3	1	C3
m=length(kunci);	C3	1	C3
if n>m	C5	1	C5
t=m+1;	C2	1	C2
KB=kunci;	C1	1	C1
while n>=t	C4	n	nC4
KB(t)=mod((KB(t-m)+KB(t-1)),256);	C2	6n	6nC2
t=t+1;	C2	n	nC2
end;			
else			
KB=kunci(1:n);			
end;			
data=[];	C1	1	C1
for j=1:n	C4	n	nC4
hasil=CB(j)+KB(j);	C2	n	nC2
data=[data mod(hasil,256)];	C2	3	3C2
end;			
n=length(data);	C3	1	C3
i=1;	C1	1	C1
Plain=[];	C1	1	C1
while n>i	C4	n	nC4
X=data(i);	C1	n	nC1
baris=[];	C1	n	nC1
while X~=59	C4	256n	256nC4
baris=[baris X];	C1	256n	256n
i=i+1;	C2	256n	256nC2
X=data(i);	C1	256n	256nC1
end;			
hasil=strcat(baris);	C3	n	nC3
Plain=[Plain; cellstr(hasil)];	C3	2n	2nC3
i=i+1;	C2	n	nC2
end;			
time=toc;	C1	1	C1
Td=time	C1	1	C1
